# Transgenic *Medicago truncatula* Plants That Accumulate Proline Display Enhanced Tolerance to Cadmium Stress

**DOI:** 10.3389/fpls.2022.829069

**Published:** 2022-01-26

**Authors:** Vanesa S. García de la Torre, Teodoro Coba de la Peña, M. Mercedes Lucas, José J. Pueyo

**Affiliations:** ^1^Instituto de Ciencias Agrarias, Consejo Superior de Investigaciones Cientiíficas (ICA-CSIC), Madrid, Spain; ^2^Centro de Estudios Avanzados en Zonas Áridas (CEAZA), La Serena, Chile

**Keywords:** Δ^1^-pyrroline-5-carboxylate synthetase, P5CS, proline, *Medicago truncatula*, cadmium, phytochelatin biosynthesis, antioxidant response

## Abstract

Cadmium (Cd) accumulation in agricultural soils constitutes a serious problem for crop yields and food safety. It is known that proline (Pro) can rapidly accumulate in plant tissues in response to abiotic stress. To analyze the potential protective effect of Pro accumulation against Cd toxicity, we compared the response to Cd stress of wild-type (WT) *Medicago truncatula* and a transgenic line that we had previously obtained and characterized (p18), which expressed the Δ*^1^-pyrroline-5-carboxylate synthetase* gene from *Vigna aconitifolia* (*VaP5CS*), and accumulated high Pro levels. Cadmium significantly reduced germination of WT seeds compared to p18 seeds, and seedling relative root growth, a valid indicator of metal tolerance, was significantly higher for p18 than WT seedlings. We analyzed the relative expression of genes related to Pro metabolism, phytochelatin biosynthesis. antioxidant machinery, and NADPH recycling, which are relevant mechanisms in the response to Cd stress. They presented differential expression in the seedlings of both genotypes both under control conditions and under Cd stress, suggesting that the Cd response mechanisms might be constitutively activated in the transgenic line. Pro accumulation promoted higher survival, enhanced growth performance, and minor nutrient imbalance in transgenic p18 plants compared to WT plants. These facts, together with the recorded gluthatione levels, lipid peroxidation and antioxidant enzyme activities strongly suggested that *VaP5CS* expression and Pro accumulation conferred enhanced Cd tolerance to *M. truncatula* p18 plants, which was likely mediated by changes in Pro metabolism, increased phytochelatin biosynthesis and a more efficient antioxidant response. Moreover, p18 roots accumulated significantly higher Cd amounts than WT roots, while Cd translocation to the aerial part was similar to WT plants, thus suggesting that high Pro levels increased not only Cd tolerance, but also Cd phytostabilization by rhizosequestration.

## Introduction

The occurrence of polluted soils with trace metal elements, also known as heavy metals (Pourret et al., 2020), is an increasing agricultural problem due to industry-derived metal contamination and to the use of soil amendments and agrochemicals. Cadmium (Cd) presents a high mobility and bioavailability and it is extremely toxic for all living organisms (Clemens et al., 2013). Plants growing in Cd-polluted soils constitute its main entry into the food chain, and present a serious threat for animal and human health (Peralta-Videa et al., 2009). Therefore, the identification of Cd-tolerant cultivars that do not translocate the metal to the edible parts of the plant becomes a priority in global food security.

Multiple studies in different plant species report that Cd is highly phytotoxic and can cause germination inhibition, nutritional imbalance, growth reduction and plant death, among other detrimental effects (Sanità di Toppi and Gabbrielli, 1999; Benavides et al., 2005; Seneviratne et al., 2019; El Rasafi et al., 2020; García de la Torre et al., 2021). Cd contributes to the generation of reactive oxygen species (ROS) (Sharma and Dietz, 2009; Romero-Puertas et al., 2019), which are considered the main source of the damage to plant tissues (Cuypers et al., 2010). Enzymatic and non-enzymatic antioxidant defense systems participate in ROS scavenging. The primary defense against ROS includes the superoxide dismutase (SOD) enzymes that catalyze the dismutation of O_2_^–^ (superoxide) to O_2_ and H_2_O_2_. Then, catalases and peroxidases can detoxify H_2_O_2_. Levels of the antioxidant metabolites ascorbate, glutathione, NADPH and the enzymes of the ascorbate-glutathione cycle are also of pivotal importance to maintain the redox state of the cell. NAPDH is considered a limiting metabolite in the plant antioxidant capacity because many ROS-detoxifying reactions are NADPH-dependent (Foyer and Noctor, 2011). This enzyme cofactor and reducing agent can be produced in different pathways, including enzymatic reactions catalyzed by isocitrate dehydrogenase (ICDH), glucose 6-phosphate dehydrogenase (G6PDH) or 6-phosphogluconate dehydrogenase (6PGDH), which are induced in response to oxidative stress caused by different factors, including Cd stress (Marino et al., 2007, 2013; Pérez-Chaca et al., 2014). The Cd-induced gene expression of these enzymes might be key for Cd tolerance in some *M. truncatula* cultivars (García de la Torre et al., 2021). Another plant response to Cd stress is the synthesis of phytochelatins (PCs) by phytochelatin-synthases (PCS), using glutathione as a substrate. It has been suggested that PCs are important for Cd chelation, detoxification and tolerance (Schat et al., 2002; Sobrino-Plata et al., 2009; Ahmad et al., 2019).

Proline (Pro) is a compatible solute accumulated under several environmental stresses in plants, such as osmotic stress and metal-induced stress, and increased levels of this amino acid have been shown to correlate with enhanced stress tolerance in several plants (Sharma and Dietz, 2006; Hayat et al., 2012; Hossain et al., 2015). The role of Pro in drought and salt stress tolerance has been widely discussed (Hayat et al., 2012; El Moukhtari et al., 2020); however, not so much is known about the contribution of Pro to Cd tolerance in plants. Pro functions as a mediator in osmotic adjustment (Ben Ahmed et al., 2011), and contributes to the protection of membranes, enzymes and proteins under various stresses (Hayat et al., 2012; Singh et al., 2015). Furthermore, Pro contributes to maintain redox homeostasis by scavenging free radicals and ROS (Sharma and Dietz, 2006; Hoque et al., 2008; Aggarwal et al., 2011). Oxidation of Pro by proline dehydrogenase (ProDH) and proline-5-carboxylate dehydrogenase (P5CDH) enhances the reducing potential of mitochondria during stress (Szabados and Savouré, 2010). Pro can also act as a metabolic signal that controls cellular homeostasis in response to changing environmental conditions (see review by Alvarez et al., 2021).

In plants, Pro is synthesized from glutamic acid (Glu) or from ornithine (Orn), depending on the availability of the substrates. Under stress conditions, Pro was proposed to be synthesized through the Glu pathway, which involves Δ^1^-pyrroline-5-carboxylate reductase (P5CR) and Δ^1^-pyrroline-5-carboxylate synthetase (P5CS) activities (Delauney and Verma, 1993; Mansour and Ali, 2017; Funck et al., 2020). The Orn pathway, which involves ornithine δ-aminotransferase (OAT) and P5CR, has been described as an alternative pathway that is considered non-essential for Pro biosynthesis (Funck et al., 2008), although it seems to be the predominant pathway in legumes (Abdelgawad et al., 2015). It has been reported that transgenic plants overexpressing Pro biosynthetic pathway genes display osmotic and oxidative stress tolerance (see review by Kumar et al., 2015).

Legume crops are capable to grow in nutrient-poor soils as they do not depend on nitrogen fertilization (Coba de la Peña and Pueyo, 2012). The model plant *Medicago truncatula* is related to alfalfa (*M. sativa*), it constitutes a source for genetic improvement and an optimal model to elucidate metal tolerance mechanisms. This forage legume presents a high biomass production and good soil coverage. *M. truncatula* cultivars with enhanced Cd tolerance and metal accumulation restricted to roots have the potential to be used as a forage crop in mildly Cd-contaminated soils (García de la Torre et al., 2021).

In a previous work, we generated a *M. truncatula* transgenic line, namely p18, that expressed the Δ*^1^-pyrroline-5-carboxylate synthetase* gene from *Vigna aconitifolia* (*VaP5CS*), accumulated high Pro levels and displayed enhanced tolerance to osmotic stress (Verdoy et al., 2006). The aim of the present work was to elucidate the role of Pro in Cd tolerance in this transgenic line. We evaluated Cd tolerance and determined the expression profiles of relevant genes involved in Pro metabolism, PC biosynthesis, antioxidant machinery and NADPH recycling in wild type (WT) and transgenic (p18) *M. truncatula* plants at the seedling stage. We also comparatively assessed Cd effect on germination, survival, metal accumulation and nutritional status at longer exposure times. Some markers related to Cd-induced antioxidant defenses were also analyzed. Our results indicated that the expression of *VaP5CS*, and the consequent Pro accumulation, remarkably enhanced the capacity of *M. truncatula* transgenic plants to cope with Cd stress.

## Materials and Methods

### Plant Material and Germination Assay

Seeds of wild-type *Medicago truncatula* Gaertn. R-108-1 (c3) and transgenic p18 *M. truncatula*, expressing the *Vigna aconitifolia* Δ*^1^-pyrroline-5-carboxylate synthetase* gene (*VaP5CS*) (Verdoy et al., 2006), were scarified, sterilized with 10% bleach for 15 min, rinsed (4 × 20 min) and imbibed in sterile water at 4°C. For the germination assay in the presence of Cd, WT and transgenic p18 *M. truncatula* seeds were transferred to Petri dishes containing 1% agar supplemented with 0, 0.5, 1.0, or 1.5 mM CdCl_2_ (25/19°C, 16/8 h). Germination percentage was evaluated after 7 days in darkness. Three biological replicates, each containing 12 seeds per genotype and treatment, were assayed.

### Cadmium Stress at the Seedling Stage

For this and all subsequent experiments, scarified, sterilized and imbibed seeds (as described above) were germinated on 1% agar Petri dishes (25/19°C, 16/8 h) for 48 h in darkness. Seedlings were acclimatized to the different growth systems with Hoagland solution (pH 5.4) prior to Cd treatment. All experiments were performed at 180 μmol photon m^–2^ s^–1^ light intensity and 25/19°C, 16/8 h photoperiod.

Germinated WT and transgenic p18 *M. truncatula* seedlings were transferred to a glass box hydroponic culture system (García de la Torre et al., 2013, 2021) for 24 h before Cd treatment was applied, and seedlings were subsequently exposed to 0 or 10 μM CdCl_2_ for 48 h. Seedling relative root growth (RRG), which is a proven indicator of metal tolerance (Sledge et al., 2005; García de la Torre et al., 2013, 2021) was determined. The CdCl_2_ concentration and time of exposure were determined in a previous study as optimal to identify Cd-sensitive and Cd-tolerant cultivars (García de la Torre et al., 2021). Two biological replicates, each containing 10 seedlings per genotype and treatment (control or Cd), were performed.

### Gene Expression Profiles

Germinated WT and transgenic p18 *M. truncatula* seedlings were acclimatized for 72 h in a glass box hydroponic culture and then exposed to Cd stress. Seedlings were exposed to 0 or 50 μM CdCl_2_ for 12 h. Roots and shoots were separately frozen in liquid nitrogen and stored at −80°C until use for RNA isolation and RT-qPCR analysis. Four biological replicates, each containing 10 seedlings per genotype and treatment, were assayed. Total RNA was isolated using the TRIZol (Invitrogen) reagent and treated with RNase-free DNase I (Thermo Scientific). Determination of RNA concentration and integrity, and reverse transcription were performed as detailed by García de la Torre et al. (2021). qPCR analyses were performed with a 7300 Real-Time PCR Sequence Detection System (PE Applied Biosystems), as described (García de la Torre et al., 2021). Fold-changes ≥ 2 were considered as significant. Specific primers were designed using the Primer3 software^1^ (Supplementary Table 1). *MtACTIN11* was used as a housekeeping gene (García de la Torre et al., 2021). Four biological replicates, each containing 10 seedlings per cultivar and treatment, were analyzed.

### Plant Growth and Survival Assays

Scarified, sterilized, imbibed WT and transgenic p18 *M. truncatula* seeds were germinated on 1% agar in Petri dishes (25/19°C) for 48 h in the dark. Germinated seedlings were acclimatized for 7 days in pots containing vermiculite (350 mL) and then watered with Hoagland nutrient solution (pH 5.4) containing 0, 0.1, 0.2, 0.4, 0.6 or 1.0 mM CdCl_2_ every 48 h. Plants were grown at 180 μmol photon m^–2^ s^–1^ light intensity, 25/19°C and 16/8 h photoperiod. After 32 days, the survival percentage was evaluated. Plants growing at 0 and 100 μM CdCl_2_ were selected for further analyses. 100 μM CdCl_2_ was chosen as a concentration that had an effect on plant growth, yet did not result in plant death, so that the differential response of WT and p18 plants could be analyzed. Shoots and roots of sixteen plants were independently frozen in liquid nitrogen and stored at –80°C for quantification of Pro and glutathione, lipid peroxidation, and SOD and CAT activities. Ten plants were used to analyze shoot and root weights, root length, number of leaves and chlorophyll content. Relative parameters (RX) were calculated as: RX = (ΔParameter X_Cd_/ΔParameter X_Control_) × 100. The same plants were used for determination of Cd and nutrients contents.

### Cadmium and Nutrient Contents

At harvest, after 32-day treatment, plant tissues were washed with 10 mM Na_2_EDTA and then rinsed with distilled water. Shoots and roots were stove-dried at 60°C. Pools of two to three plants per genotype and treatment were digested with nitric-perchloric acid (7:3). Elemental analyses were performed using an ICP-OES (Perkin Elmer Optima 4300 DV). Three to five replicates (pools) per genotype and treatment were analyzed.

### Determination of Free Proline, Glutathione, Lipid Peroxidation and Antioxidant Enzyme Activities

Shoots and roots of WT and transgenic p18 *M. truncatula* plants were ground in liquid nitrogen and homogenized in 5% (w/v) sulfosalicylic acid (0.2 g FW mL^–1^). Homogenates were centrifuged (15,000 *g*, 5 min, 4°C), and the supernatant was used for free Pro determination as previously described (Bates et al., 1973). The reaction mixture included the supernatant, acid ninhydrin and glacial acetic acid (1:1:1). The acid ninhydrin solution was prepared in the dark by mixing 30 mL glacial acetic acid, 20 mL 6 M phosphoric acid and 1.25 g ninhydrin. The reaction mixture was boiled for 1 h and the reaction was stopped on ice. Proline-ninhydrin chromophore complex was extracted using 0.6 mL toluene. Absorbance of the organic phase was determined at 520 nm. Content of Pro was determined with a calibration curve performed with L-proline. Four biological replicates per genotype and treatment were analyzed.

Total glutathione (GSH + GSSG) and reduced glutathione (GSH) were determined as we previously described (García de la Torre et al., 2021) in roots and shoots of WT and transgenic *M. truncatula* p18 plants grown at 0 or 100 μM CdCl_2_ for 32 days. Four biological replicates per genotype and treatment were analyzed.

Malondialdehyde (MDA) determination was carried out using the thiobarbituric acid method (Singh et al., 2007). Plant tissue was ground in liquid nitrogen and homogenized in 0.1% TCA solution and MDA was determined as described by Redondo et al. (2009). Four biological replicates per genotype and treatment were analyzed.

The enzyme activities were assayed in roots and shoots of WT and transgenic p18 *M. truncatula* plants grown in the presence of 0 or 100 μM CdCl_2_ for 32 days. Plant tissue was ground in liquid nitrogen and homogenized (García de la Torre et al., 2021). SOD and CAT activities were determined as described (Redondo et al., 2009). Total protein content in the homogenates was quantified in 1:5 dilutions at 595 nm with the Bradford protein assay (BioRad) using BSA as a standard. The enzymatic activities were calculated in units per mg of protein. One unit of SOD is the amount of enzyme that will inhibit the rate of reduction of cytochrome c by 50% in a coupled system, using xanthine and xanthine oxidase at pH 7.8 at 25°C. One catalase unit will decompose 1.0 μmole of H_2_O_2_ per minute at pH 7.0 at 25°C. Four biological replicates per genotype and treatment were analyzed.

### Statistical Analyses

The IBM SPSS Statistics 20 software (SPSS Inc., Chicago, IL, United States) was used. Root length and the relative growth parameters were analyzed by ANOVA (*p* < 0.05). Cadmium and nutrient contents were analyzed by ANOVA (*p* < 0.05, Tukey HSD). The germination and survival percentages were analyzed by Pearson’s Chi-squared test (χ^2^) (*p* < 0.05). The glutathione and Pro contents, SOD and CAT activities and lipid peroxidation were analyzed by ANOVA (*p* < 0.05) and the Tukey HSD test was used for pair-wise comparisons.

## Results

### Expression of *VaP5CS* in *M. truncatula* Reduces the Negative Effects of Cadmium Stress on Germination and on Root Growth at the Seedling Stage

**FIGURE 1:**
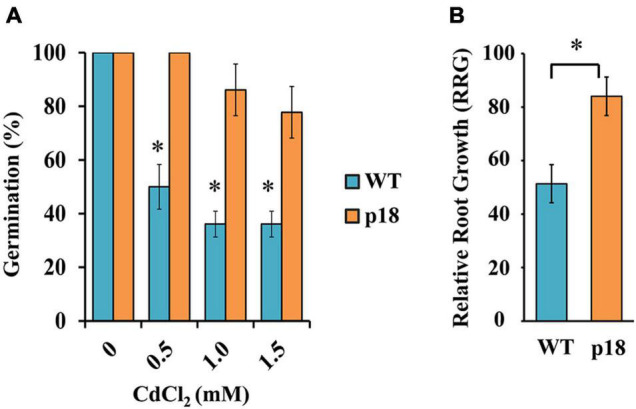
Effects of cadmium on germination and seedling relative root growth of *M. truncatula* WT and p18 lines. **(A)** Germination percentage of *M. truncatula* WT and transgenic p18 seeds at different CdCl_2_ concentrations for 7 days. * Indicates significant differences between control and Cd treatment (Chi-squared (χ^2^), *p* < 0.05, *n* = 3 replicates per treatment, each containing 12 seeds). **(B)** Relative root growth (RRG) of *M. truncatula* WT and transgenic p18 seedlings in the presence of Cd (40 μM CdCl_2_, 48 h). * Indicates significant differences between genotypes, one-way ANOVA, *p* < 0.05, *n* = 10. Bars indicate standard deviation.

As a first approach to determine whether Pro accumulation had an effect on *M. truncatula* tolerance to Cd, we compared the effect of Cd on seed germination. WT and p18 seeds were germinated in the presence of increasing CdCl_2_ concentrations. Cadmium induced a strong and significant decrease in the germination percentage of WT seeds at all tested Cd concentrations. A slight decrease could be observed in p18 seed germination, but Cd had a much lesser effect (Figure 1A).

To examine how Pro accumulation influenced Cd tolerance, we compared the relative root growth (RRG) of *M. truncatula* p18 and WT seedlings growing in a miniaturized hydroponic system. RRG at the seedling stage is considered a valid indicator of metal tolerance in plants (Sledge et al., 2005; García de la Torre et al., 2013, 2021). These conditions were previously described as adequate to identify Cd-sensitive and Cd-tolerant cultivars (García de la Torre et al., 2021). Upon Cd treatment, both genotypes displayed a significant decrease in root growth compared to control conditions (Figure 1B). However, whereas Cd affected more severely WT seedlings (RRG ≈ 50%), p18 seedlings root growth was reduced by about 17% in the presence of Cd. These results suggested that *VaP5CS* expression and the consequent Pro accumulation led to increased tolerance to Cd stress in p18 plantlets.

### Pro Metabolism, Phytochelatin Biosynthesis, and Antioxidant Response Related Genes Display Different Relative Expression in p18 and Wild-Type Seedlings

**FIGURE 2:**
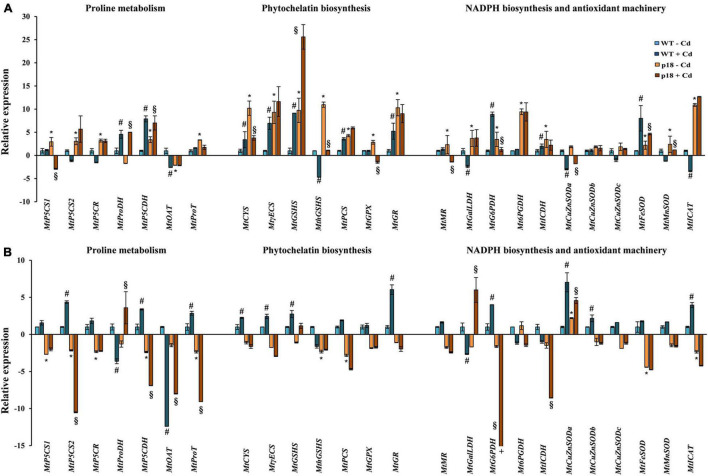
Transcript accumulation of genes involved in Pro metabolism, PC biosynthesis, antioxidant machinery and NADPH biosynthesis in *M. truncatula* WT and transgenic p18 seedlings roots **(A)** and shoots **(B)** grown in the absence or presence of 50 μM CdCl_2_ for 12 h. Data are mean of four biological replicates (10 seedlings per replicate). * Indicates significant differences (fold change ≥ 2) between untreated WT and p18 seedlings; #, significant differences between untreated and Cd-treated WT seedlings; §, significant differences between untreated and Cd-treated p18 seedlings; +, the actual value is –37.8. Abbreviations: *MtP5CS1*: *M. truncatula*Δ^1^-pyrroline-5-carboxylate synthetase 1; *MtP5CS2*: Δ^1^-pyrroline-5-carboxylate synthetase 2; *MtP5CR*: pyrroline-5-carboxylate reductase; *MtP5CDH*: Δ^1^-pyrroline-5-carboxylate dehydrogenase; *MtProDH*: proline dehydrogenase; *MtOAT*: ornithine δ-aminotransferase; *MtProT*: proline transporter; *MtCYS*: cysteine synthase; *MtECS*: γ-glutamylcysteine synthetase; *MtGSHS*: glutathione synthetase; *MthGSHS*: homoglutathione synthetase; *MtPCS*: phytochelatin synthase; *MtGPX*: glutathione peroxidase; *MtGR*: glutathione reductase; *MtMR*: monodehydroascorbate reductase; *MtGalLDH*: galactono-1, 4-lactone dehydrogenase; *MtG6PDH*: glucose-6-phosphate dehydrogenase; *Mt6PGDH*: 6-phosphogluconate dehydrogenase; *MtICDH*: isocitrate dehydrogenase; *MtCuZnSODa*, *MtCuZnSODb*, *MtCuZnSODc*: cooper/zinc superoxide dismutases; *MtFeSOD: ferric SOD; MtMnSOD*: manganese SOD; *MtCAT*: catalase.

The expression of *VaP5CS* and the consequent Pro accumulation, influenced the transcriptional response of several important genes related to Pro metabolism, phytochelatin (PC) biosynthesis, antioxidant machinery and NADPH biosynthesis both in the absence and the presence of Cd (Figure 2). In the absence of Cd, most tested genes showed significantly higher expression in p18 than in WT roots. *MtOAT* showed significantly lower expression in p18 than in WT roots, *MtProDH* expression decreased in p18 roots, although non-significantly, and *MtCuZnSODs* expression did not show any significant differences (Figure 2A). On the contrary, most genes showed a lower expression in p18 than in WT shoots. A significant downregulation was observed for most genes involved in biosynthesis, transport and degradation of Pro (*MtPC5CS1*, *MtPC5CS2*, *MtP5CR*, *MtP5CDH* and *MtProT*), for *MthGSHS*, *MtPCS* involved in PC biosynthesis, and for antioxidant response related *MtFeSOD* and *MtCAT*. Only *MtCuZnSODa* showed significantly higher expression in p18 than in WT shoots. Most genes involved in glutathione, ascorbate and NADPH biosynthesis showed no significant changes in expression between p18 and WT shoots (Figure 2B).

In WT roots, Cd stress caused an upregulation of *MtProDH*, *MtP5CDH*, of most of the genes involved in GSH and PCs biosynthesis, and several genes involved in NADPH biosynthesis and antioxidant response (*MtG6PDH*, *MtICDH* and *MtFeSOD*). *MtOAT*, *MthGSHS, MtGalLDH*, *MtCuZnSODa* and *MtCAT* showed lower expression in WT roots under Cd stress (Figure 2A). In WT shoots, most genes were upregulated upon Cd exposure, and this upregulation was significant for *MtP5CS2*, *MtP5CDH*, *MtProT*, *MtCYS*, *MtECS*, *MtGSHS*, *MtGR*, *MtG6PDH*, *MtCuZnSODa* and *MtCuZnSODb*, and *MtCAT*. Only a few genes (*MtProDH, MtOAT* and *MtGalLDH*) were significantly downregulated upon Cd exposure in WT shoots (Figure 2B). On the other hand, only a few genes (*MtProDH*, *MtP5CDH, MtGSHS* and *MtFeSOD*) were significantly upregulated in p18 roots upon Cd stress in comparison with untreated p18 roots, whereas a significant downregulation was observed for *MtP5CS1*, *MtCYS*, *MthGSHS, MtGPX, MtMR*, *MtG6PDH*, *MtCuZnSODa* and *MtMnSOD* expression in Cd-treated p18 roots (Figure 2A). Cd stress led to significantly increased expression of *MtProDH, MtGalLDH* and *MtCuZnSODa* in p18 shoots, and to significantly reduced expression of several genes related to Pro metabolism (*MtP5CS2*, *MtP5CDH*, *MtOAT*, *MtProT*), and NADPH biosynthesis (*MtG6PDH* and *MtICDH*). Genes related to the biosynthesis of glutathione and PC were not affected upon Cd exposure in p18 shoots (Figure 2B).

### Proline Accumulation Enhances Cadmium Tolerance in Transgenic *M. truncatula* p18 Plants, and Affects the Nutritional Status of Cd-Stressed Plants

**FIGURE 3:**
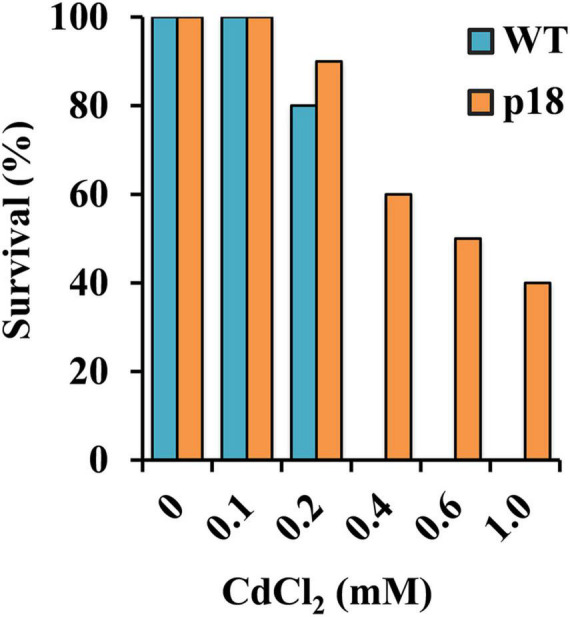
Survival percentage of WT and p18 plants upon exposure to different CdCl_2_ concentrations (*n* = 30). The germination percentages were analyzed by Pearson’s Chi-squared test (χ2) (*p* < 0.05).

**FIGURE 4:**
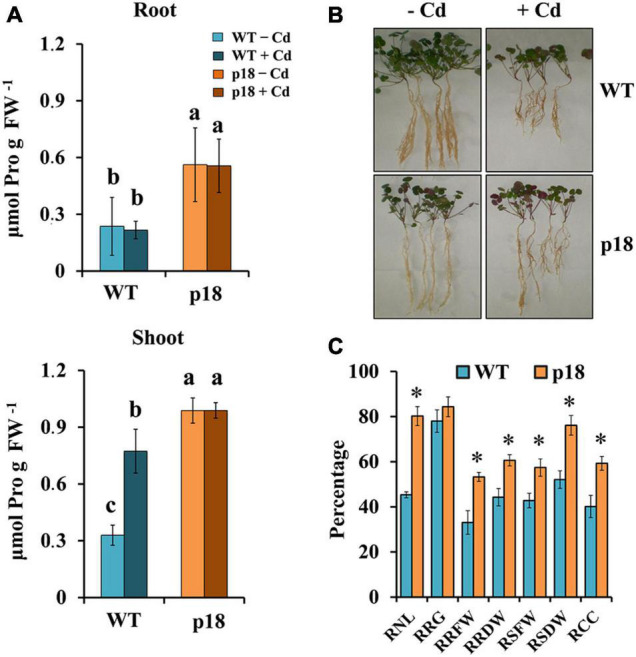
Effects of cadmium treatment on *M. truncatula* WT and transgenic p18 plants after 32 days of growth in pots in the presence of 0 or 100 μM CdCl_2_. **(A)** Pro content in roots (upper panel) and shoots (lower panel). Different letters denote significant differences between lines and treatments in roots or shoots, Tukey HSD, *p* < 0.05, *n* = 4. **(B)** Representative image of *M. truncatula* WT and p18 plants grown for 32 days in the absence or presence of 100 μM CdCl_2_. **(C)** Relative growth parameters of Cd-treated *M. truncatula* WT and p18 plants calculated as percentages of the values recorded for plants grown in the absence of Cd. Bars indicate standard error. * Indicates significant differences between genotypes (ANOVA, *p* < 0.05, *n* = 10). RNL, relative number of leaves; RRG, relative root growth; RRFW, relative root fresh weight; RRDW, relative root dry weight; RSFW, relative shoot fresh weight; RSDW, relative shoot dry weight; RCC, relative chlorophyll content.

Plants of both genotypes grown in vermiculite were watered with nutrient solution containing different CdCl_2_ concentrations for 32 days. Survival was negatively affected in both genotypes at concentrations of CdCl_2_ higher than 100 μM. WT plants did not survive exposure to 400 μM CdCl_2_ or to higher concentrations. The transgenic line was affected, but displayed notable survival percentages when exposed to 400 μM CdCl_2_ and higher concentrations up to 1 mM CdCl_2_ (Figure 3). To further characterize the role of Pro accumulation in the *M. truncatula* transgenic line Cd tolerance, and to determine the differences between WT and p18 plants after a longer exposure to Cd, we selected plants subsets grown in the absence of Cd or in the presence of 100 μM CdCl_2_, a concentration that did not affect survival of WT or p18 plants. Pro content, growth parameters, Cd accumulation and nutrients contents were measured in roots and shoots. The content of Pro was significantly higher in p18 compared to WT plants, both in roots and shoots in the absence or presence of Cd (Figure 4A), due to expression of *VaP5CS* gene. However, Cd induced a significant increase in Pro accumulation in shoots of WT plants only (Figure 4A). Differences in plant growth between WT and transgenic p18 plants were observed after Cd stress (Figure 4B). Relative growth parameters calculated as percentages of the values recorded for plants grown in the absence of Cd were significantly higher for p18 than for WT plants subjected to Cd stress, including the relative number of leaves (RNL), relative root fresh weight (RRFW), relative root dry weight (RRDW), relative shoot fresh weight (RSFW), relative shoot dry weight (RSDW) and relative chlorophyll content (RCC). The difference was not significant for relative root growth (RRG) (Figure 4C).

Regarding metal accumulation and translocation to the aerial part, Cd accumulated mainly in roots and relatively small amounts were translocated to the aerial part of the plants (Table 1). Transgenic p18 plants accumulated a significantly higher content of Cd in roots (about 1.5-fold). In shoots, differences in Cd content, while still significant, were much smaller. In consequence, the translocation factor was significantly higher in WT plants (Table 1).

**TABLE 1:** Cadmium content in roots and shoots of WT and transgenic p18 *M. truncatula* plants.

	[Cd] mg/kg		Translocation factor%
	Root	Shoot	
WT	893.56 ± 295.28 b	62.03 ± 8.84 b	7.94 ± 3.60 a
p18	1327.51 ± 153.19 a	70.13 ± 3.60 a	5.37 ± 0.88 b

*Plants were grown in pots containing vermiculite with 0 or 100 μM CdCl_2_ for 32 days.*

*Means ± SD are shown.*

*Different letters denote significant differences between WT and p18 plants (Tukey HSD, p < 0.05, n = 3–5).*

The content of macro and micronutrients did not show striking differences between WT and p18 plants grown under control conditions for 32 days (Table 2). In roots, only S content was significantly lower in p18 than in WT plants, and Zn and Cu contents were significantly higher in p18 compared to WT roots. In shoots, p18 plants showed significantly lower contents of K and P than WT plants; while Ca, Na and Zn contents were significantly higher in p18 than in WT shoots.

**TABLE 2:** Macro and micronutrients contents in roots and shoots of WT and transgenic p18 *M. truncatula* plants.

		Root		Shoot	
	CdCl_2_ (μM)	WT	p18	WT	p18
**Macronutrients (mg kg^–1^)**					
K	0	46,500	49,442	62,701 a	53,112 b
	100	58,797 *	55,182 *	51,769 *	51,908
Ca	0	1,293	1,384	3,634 b	4,267 a
	100	1,771 *	1,675 *	3,430	3,177 *
Na	0	2,230	2,235	858 b	1,028 a
	100	3,147 *	2,956 *	1,292 *b	1,775 *a
Mg	0	10,069	10,458	11,246	12,214
	100	9,729 *	8,113 *	13,276 *a	11,418 b
P	0	1,210	1,077	2,128 a	1,953 b
	100	741 *b	1,233 a	1,898 *	1,870
S	0	5,781 a	2,610 b	4,240	4,423
	100	1,796 *	857 *	4,436 a	2,198 *b
**Micronutrients (mg kg^–1^)**					
Fe	0	943	1,104	70	90
	100	445 *	696 *	73	75
Mn	0	183	233	37	40
	100	162 a	88 *b	47	39
Zn	0	20 b	33 a	11 b	16 a
	100	26 *b	35 *a	5 *	7 *
Cu	0	31 b	56 a	16	17
	100	63 *b	87 *a	12 *b	19 a

*Plants were grown in pots containing vermiculite with 0 or 100 μM CdCl_2_ for 32 days.*

*An asterisk indicates significant differences between treatments; different lower-case letters indicate significant differences between WT and p18 plants (ANOVA, p < 0.05, n = 3–5).*

In WT roots, the Cd treatment promoted a significant increase of the contents of K, Ca, Na, Zn and Cu, and a significant decrease in Mg, P, S and Fe contents. The effect of Cd on p18 roots was similar to that observed in WT roots, with the exceptions of P (that was not significantly affected by the Cd treatment) and Mn (that displayed a significant decrease upon Cd treatment); Mn content was significantly lower than in treated p18 than in treated WT roots. Zn and Cu contents were significantly higher in p18 than in WT roots, both in the absence and in the presence of Cd.

In WT shoots, Cd treatment induced a significant increase in the contents of Na and Mg, and a significant decrease in the contents of K, P, Zn and Cu. In Cd-treated p18 shoots, a significant increase in Na content was observed, but no significant changes were observed in the contents of Mg. Na levels remained significantly higher than those observed for Cd-treated WT shoots, and the opposite was observed for Mg levels. Significant decreases in the amounts of Ca, S and Zn were observed in p18 shoots exposed to Cd; Ca levels remained at similar levels than those observed in WT shoots upon Cd treatment, and S levels were significantly lower than those observed in Cd-treated WT shoots. The contents of K, P and Cu were not affected by the Cd treatment in p18 shoots, but Cu levels were significantly higher than those in Cd-treated WT shoots.

### Wild-Type and Transgenic p18 *M. truncatula* Plants Display Differential Antioxidant Responses to Cadmium Stress

To investigate whether the antioxidant defense was affected in the transgenic plants, we analyzed roots and shoots of WT and p18 plants grown for 32 days in presence of 0 or 100 μM CdCl_2_. The contents of oxidized (GSSG) and reduced glutathione (GSH), and the GSH/GSSG ratio, lipid peroxidation, and the SOD and CAT enzyme activities were determined.

**FIGURE 5:**
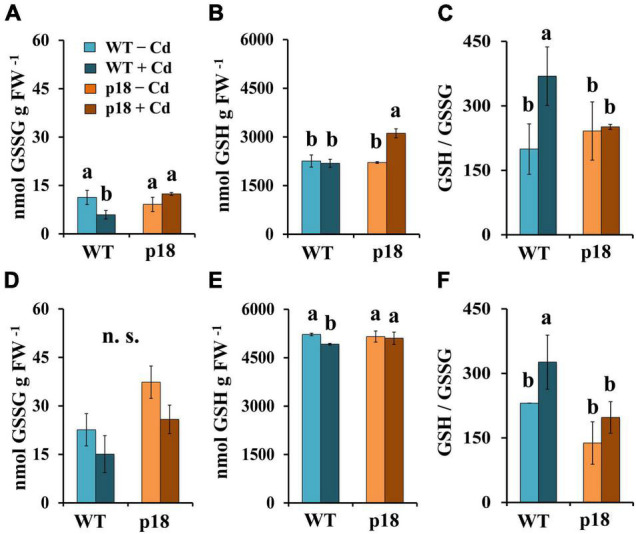
Cadmium effect on the levels of oxidized gluthathione (GSSG), reduced glutathione (GSH) and GSH/GSSG ratio in *M. truncatula* WT and transgenic p18 roots **(A–C)** and shoots **(D–F)**. Plants were grown in pots with 0 or 100 μM CdCl_2_ for 32 days. Bars indicate standard deviation. Different letters denote significant differences, Tukey HSD, *p* < 0.05, *n* = 4; n.s. not significant.

No significant differences in GSSG or GSH contents were observed when comparing WT and p18 plants in absence of Cd, neither in roots nor in shoots. A significant decrease in GSSG levels was observed in WT roots upon Cd exposure (Figure 5A), and a significant increase in GSH levels was observed in roots of Cd-treated transgenic p18 plants (Figure 5B). In shoots, GSSG levels were not significantly affected by the Cd treatment in either genotype (Figure 5D). A small, yet significant decrease in GSH levels was observed in Cd-treated WT shoots, whilst no significant changes were observed in p18 shoots (Figure 5E). Significant increases of the GSH/GSSG ratio were observed in WT roots (Figure 5C) and shoots (Figure 5F) upon Cd exposure, while no significant differences for this parameter were observed between control and Cd-treated p18 plants (Figures 5C,F).

**FIGURE 6:**
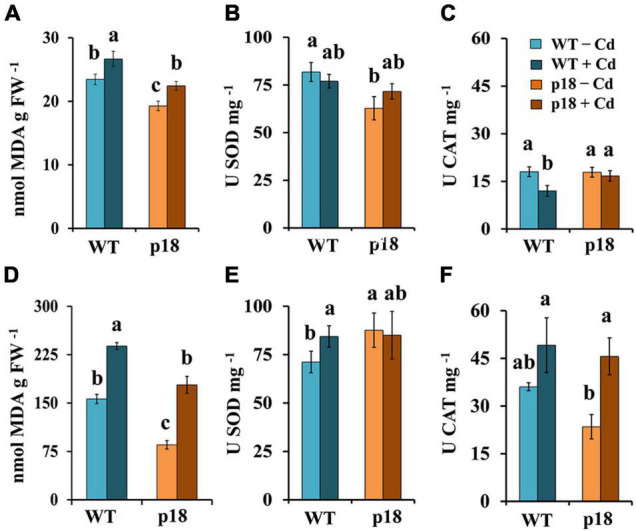
Lipid peroxidation (MDA), superoxide dismutase and catalase activities in *M. truncatula* WT and p18 roots **(A–C)** and shoots **(D–F)**. Plants were grown in pots with 0 or 100 μM CdCl_2_ for 32 days. Bars indicate standard deviation. Different letters denote significant differences, Tukey HSD, *p* < 0.05, *n* = 4.

The oxidative damage was estimated by measuring lipid peroxidation (as MDA content). In the absence of Cd, MDA levels in roots and shoots of p18 plants were significantly lower than those in roots and shoots of WT plants (Figures 6A,D). Cadmium exposure promoted a significant increase of MDA levels in roots and shoots of both genotypes; however, MDA levels in Cd-treated p18 roots and shoots remained significantly lower than those in Cd-treated WT roots and shoots, respectively (Figures 6A,D). SOD and CAT enzymatic activities were determined. In the absence of Cd, SOD activity levels were significantly higher in WT roots compared with those in p18 roots (Figure 6B), and the opposite was observed in shoots (Figure 6E). Upon Cd treatment, SOD activity was significantly induced in WT shoots, reaching to similar values to those observed in p18, which were not significantly affected by Cd (Figure 6E). Catalase activity was similar in WT and p18 roots (Figure 6C) in the absence of Cd, and it was higher, although non-significantly in WT than in p18 shoots (Figure 6F). Exposure to Cd induced a significant decrease of catalase activity in WT roots (Figure 6C), and an increase in both WT and p18 shoots that was statistically significant for p18 (Figure 6F).

## Discussion

Plants accumulate Pro under a variety of abiotic stresses including salinity, drought and heavy metal-induced stress (Sharma and Dietz, 2006; Hayat et al., 2012). This amino acid functions in plants as a signaling molecule (Singh et al., 2017), as a mediator in osmotic adjustment (Ben Ahmed et al., 2011), a stabilizer of sub-cellular structures (Singh et al., 2015), and as a ROS scavenger (Aggarwal et al., 2011). However, little is known about the role of Pro in plants under Cd stress.

To obtain a deeper insight into the role of Pro in Cd tolerance in *M. truncatula*, we used a transgenic line, p18, that expresses the Δ^1^-pyrroline-5-carboxylate synthetase gene from *Vigna aconitifolia* (*VaP5CS*) and accumulates high Pro levels (Verdoy et al., 2006). In our previous work we used two different transgenic lines, p2 and p18, which behaved in an identical manner, only p2 showed relatively higher *VaP5CS* transcript and Pro accumulation than p18. However, p2 germination was extremely poor and we decided to use only p18 in the present study. We evaluated Cd tolerance, expression profiles of relevant genes involved in Pro biosynthesis and response to Cd stress, as well as growth performance, Cd accumulation, and nutritional status in WT *M. truncatula* plants and in the transgenic line.

Expression of the *VaP5CS* transgene in wheat (Sawahel and Hassan, 2002), tobacco (Razavizadeh and Ehsanpour, 2009), chickpea (Ghanti et al., 2011), sugarcane (Guerzoni et al., 2014), rice (Kumar et al., 2010) and pigeon pea (Surekha et al., 2014) has been proven to promote high levels of Pro accumulation and, to different extents, better plant performance under salt stress. Expression of *VaP5CS* has been reported to increase Cd stress tolerance in the green algae *Chlamydomonas reinhardtii* (Siripornadulsil et al., 2002). To our knowledge, the effect on Cd tolerance of *VaP5CS* (or any other *P5CS* homolog) expression, and the consequent Pro accumulation, has not been reported in higher plants to date. In the present work, we show that expression of *VaP5CS* leads to Pro accumulation and increased tolerance to Cd stress in *M. truncatula*.

Compared with other developmental stages, germination is very sensitive to Cd stress (Rahoui et al., 2008; Seneviratne et al., 2019; El Rasafi et al., 2020). Our results indicated that the expression of *VaP5CS* allowed elevated germination in the presence of Cd concentrations that significantly decreased germination of *M. truncatula* WT seeds. Seedling relative root growth (RRG) is considered as a valid indicator of metal tolerance (Sledge et al., 2005; García de la Torre et al., 2013, 2021; Rahoui et al., 2014). The RRG assay showed that *M. truncatula* transgenic line p18 displayed significantly higher tolerance to Cd stress than WT *M. truncatula* at the seedling stage.

In general, Pro accumulation upon stress involves the Glu pathway by increased expression and/or activity of P5CS (Delauney et al., 1993; Zhen and Ma, 2009; Lei et al., 2016; Mansour and Ali, 2017; Funck et al., 2020). *MtOAT* expression was significantly decreased in shoots and roots of WT seedlings exposed to Cd, while *MtP5CS2* expression was significantly induced in shoots of WT seedlings exposed to Cd, suggesting that the Glu pathway might be the main source of Pro accumulation upon Cd stress in *M. truncatula* at the seedling stage. Nevertheless, the Orn pathway seems to play a role in Pro accumulation in adult *M. truncatula* plants under salt stress (Verdoy et al., 2006). In the absence of Cd, the roots of p18 transgenic plantlets displayed higher expression of most genes related to Pro biosynthesis and transport (*MtP5CS1*, *MtP5CS2*, *MtP5CR*, and *MtProT*), in comparison with WT plantlets.

In addition to scavenge, stabilize and detoxify ROS, Pro accumulation might lead to an activation of the antioxidant machinery and the biosynthesis of phytochelatins (PCs) (Siripornadulsil et al., 2002; Sharma and Dietz, 2006; Szabados and Savouré, 2010; Hayat et al., 2012; Ahmad et al., 2019). The plant antioxidant machinery plays a fundamental role in Cd tolerance (Terrón-Camero et al., 2020). To maintain redox homeostasis, SOD and CAT are the first enzymes to face ROS accumulation. In addition, GSH has a dual role as an antioxidant molecule and as a substrate for phytochelatin biosynthesis (Noctor et al., 2012; Sobrino-Plata et al., 2014). NAPDH-recycling enzymes play an important role in Pro biosynthesis. In plants, NADPH is key in the antioxidant defense response (Foyer and Noctor, 2011) and Cd tolerance appears to be dependent on NADPH levels in some species (León et al., 2002; Pérez-Chaca et al., 2014). Genes codifying for NAPDH-recycling enzymes have been proposed to be key in the strategy to cope Cd stress in a Cd-tolerant *M. truncatula* cultivar (García de la Torre et al., 2021). Our results showed that, in the absence of Cd, the expression of *VaP5CS* promoted enhanced expression of most tested genes related to the antioxidant machinery, PC biosynthesis and NADPH-recycling in p18 transgenic roots compared to WT roots, suggesting that p18 plants possess a constitutive capacity to better counteract Cd toxicity. In transgenic shoots, though, most genes showed lower expression than in WT shoots. Upon Cd treatment, most genes showed enhanced expression in both WT roots and shoots, while only a few genes were upregulated in the transgenic plants, suggesting that the Cd treatment was stressful for WT plants and induced a transcriptomic response, while p18 plants were less affected. Increased glutathione reductase (*GR*) expression is considered as a marker of Cd stress in plants (Romero-Puertas et al., 2007; DalCorso et al., 2008; Chou et al., 2012). Interestingly, *MtGR* expression was higher in transgenic roots than in WT roots in the absence of Cd. This elevated *MtGR* expression cannot be ascribed to stress, and it is most likely a result of Pro accumulation. When Cd treatment was applied, WT plants displayed a significant increase in *MtGR* expression, indicating a response to Cd stress, while such increase was not observed in p18 roots or shoots, suggesting that the transgenic line was less stressed by the Cd treatment.

We analyzed WT and p18 plants grown in the absence or presence of Cd for 32 days. As expected, Pro content was significantly higher in roots and shoots of the transgenic line than in WT plants. Cd treatment did not induce changes in Pro contents except for a significant increase in WT shoots, which nevertheless, remained lower than that in p18 shoots. Our results on Cd contents suggest that, due to Pro accumulation, certain mechanisms are likely triggered in p18 plants that induce Cd accumulation, mainly in roots. The enhanced expression of phytochelatin synthase (*PCS*) in p18 roots could contribute to increased Cd chelation by phytochelatins and subsequent Cd storage in the vacuole (El Rasafi et al., 2020). Other genes and proteins, such as metal transporters, phytochelatin transporters and metallothioneins might also be involved (Ismael et al., 2019), but were not the object of the present study. It has been proposed that Pro may form non-toxic complexes with Cd (Rehman et al., 2017; El Rasafi et al., 2020). It is well known that Cd causes plant nutritional imbalance by impairing the uptake and transport of several nutrients (Ismael et al., 2019; Qin et al., 2020). The decreases in Fe and Mn contents in roots under Cd stress might suggest that Fe and/or Mn transporters could be involved in Cd uptake by *M. truncatula* roots (Vert et al., 2002; Sasaki et al., 2012). Phosphorus (P) and Mg are essential elements for photosynthesis. The contents of these two elements were not impaired in the shoots of p18 plants upon Cd stress, in agreement with the higher chlorophyll content in p18 plants compared to WT plants after Cd treatment.

In plants, glutathione plays a key role as an antioxidant, chelator and a signaling molecule (Jozefczak et al., 2012). Cadmium promoted an increase of the GSH/GSSG ratio in roots and shoots of WT plants, while the transgenic p18 plants remained unaltered. This result might appear unexpected, as it would suggest a better redox balance. However, an increase of GSH might be needed to activate PC biosynthesis, which is in good agreement with the increased expression of *MtGR, MtCYS, Mt*γ*ECS, MtGSHS* and *MtPCS* observed in WT seedling roots upon Cd treatment. The expression of these genes is basally higher in p18, and their expression changes are not so marked after Cd exposure.

Cadmium stress induces oxidative damage that leads to lipid peroxidation in different plant species, including *M. truncatula* (Sobrino-Plata et al., 2009; Rahoui et al., 2017; García de la Torre et al., 2021). Lipid peroxidation, SOD and CAT activities were measured in shoots and roots of WT and p18 plants. Lipid peroxidation was significantly higher after Cd stress in both genotypes. Pro accumulation seemed to have a positive effect as lipid peroxidation remained always lower in p18 than in WT plants. Moreover, MDA content observed in both organs of p18 plants subjected to Cd stress were similar to those observed in unstressed WT plants. SOD activity was slightly, but significantly lower in p18 than in WT roots in the absence of Cd, and the opposite was observed in shoots. This higher basal SOD activity in p18 shoots might provide an intrinsic advantage, as Cd promoted a significant increase of SOD activity in WT shoots reaching to levels measured in non-stressed p18 shoots. Two of the SOD genes analyzed (*MtFeSOD, MtMnSOD*) showed higher expression in p18 than in WT roots. Only *MtCuZnSODa* displayed significantly higher expression in p18 than in WT shoots, while *MtFeSOD* showed a significantly lower expression. In presence of Cd, catalase activity decreased in WT roots and increased in WT shoots, in agreement with the changes in expression observed for *MtCAT*. While catalase activity was significantly higher in p18 roots than in WT roots upon Cd exposure, CAT activities did not seem to correspond to the variations in *MtCAT* (*MTR_3g115370*) expression. Catalase activity increased in the shoots of both genotypes in the presence of Cd, most probably as a response to ROS produced by the impaired photosynthetic activity (Foyer et al., 1994; Mittler, 2002), but again the activity values did not reflect the changes in *MtCAT* expression. These differences might be due to the presence of at least another *CAT* gene in the *M. truncatula* genome (*MTR_1386s0010*), which we were not able to amplify and might display different expression patterns.

The results presented here support the hypothesis that the expression of *VaPC5S* and the consequent high Pro accumulation contribute to alleviate Cd stress in *M. truncatula* transgenic p18 line. However, this increase in tolerance is not due exclusively to Pro accumulation, but to a Pro-induced upregulation of several important genes related to Pro metabolism, PC biosynthesis, antioxidant machinery, and NADPH-recycling in the transgenic line, which in this way becomes constitutively better equipped to cope with Cd stress. Moreover, the increased accumulation of Cd in p18 roots compared to WT roots, with only a slightly higher Cd levels in p18 than in WT shoots suggest that Pro accumulation also appears to increase the plant phytostabilization capacity through rhizosequestration.

## Data Availability Statement

The raw data supporting the conclusions of this article will be made available by the authors, without undue reservation.

## Author Contributions

VSGT, TCP, JJP, and MML designed the project and conceived the experiments. VSGT performed the experiments. TCP contributed to the experimental part. JJP, MML, and TCP supervised the experiments and corrected and wrote the final version. VSGT wrote the first draft. All authors approved the content of the manuscript.

## Conflict of Interest

The authors declare that the research was conducted in the absence of any commercial or financial relationships that could be construed as a potential conflict of interest.

## Publisher’s Note

All claims expressed in this article are solely those of the authors and do not necessarily represent those of their affiliated organizations, or those of the publisher, the editors and the reviewers. Any product that may be evaluated in this article, or claim that may be made by its manufacturer, is not guaranteed or endorsed by the publisher.

## Footnotes

1
http://bioinfo.ut.ee/primer3-0.4.0/
