# Supplementary material for: Transgenic Medicago truncatula Plants That Accumulate Proline Display Enhanced Tolerance to Cadmium Stress
Source: Front Plant Sci. 2022 Jan 26;13:829069. doi: 10.3389/fpls.2022.829069 (PMC8826176; doi:10.3389/fpls.2022.829069)
Supplement: Supplementary file 1 [file Data_Sheet_1.PDF]

Supplementary Table 1. Primer sequences used for gene expression analysis and accession numbers (Acc. N.) or locus names of genes in this study. Primer3 software (https://bioinfo.ut.ee/primer3-0.4.0/) was used for the primer design; Acc. N. and locus names were obtained from available genomic sequences at the National Center for Biotechnology Information (http://www.ncbi.nlm.nih.gov) and the *Medicago truncatula* Genome Project v4.0 (http://www.jcvi.org/medicago/).

Gene	Forward 5'-3'	Reverse 5'-3'	Acc. N. or Locus Name
MtACTIN11	GGTCCATCAATTGTCCACAG	ATGCTTCTGGCTGATTCACA	XM_003621972
VaP5CS	GCAAAAGTTGATTATCCGGCAG	TTCGAAGGTCAAGAATGATCTCC	M92276
MtP5CS1	TTAATTGAGAGAAATGATGATAATTTGC	CAATTTCCTAAGTTGGGAAGGAAAC	Medtr3g069080
MtP5CS2	CGGTAGGAGTCGAGGGCTTGTT	TTGTGTGTATAAATCATACTTCGATCG	Medtr7g063650
MtP5CR	GGGTTTCCGTGGAATACTGA	TCGAATTTTCGCTTAGAAATCA	Medtr7g090160
MtProDH	AGGGATTGGAAAGGTGAACCA	CCCTGCATTGCTCAAAGCA	Medtr7g020820
MtP5CDH	ATGATTATGGACCGGTATCAAAG	GGACATACTAGTCTCGCCGTCT	Medtr4g107940
MtOAT	GCAGTTGCCATTGCCTCACT	AGCAGACCGAGAAGCTCACC	Medtr7g451450
MtProT	ACAGAAGGGCGGCCTCTG	TTGGAGTCTACTGCAATGAGCC	Medtr3g069960
MtCYS	TCCTCTGTGCTATTTGAGTCAGTGA	AATGATTCAGGGCTCGAAAGTC	XM_003610676
MtECS	GCGTCTTGCTTTTCTTTCACATT	TTCTACCCAAAAAAGGTGGCTTAT	AF041340
MtGSHS	CATTACCATTTGAGCCTCCTGTT	TGTGGCCTCCCTTTCAATTT	XM_003626276
MthGSHS	CACATGATGAAACCAACTCATTGA	AACATGTCATGGGAGAAAGTAAAAGA	AF194421
MtPCS	ATCTCGGTGCTCCTTCCTCT	CAGAAGCAGAATTTTGGTGAC	Medtr7g097190
MtGPX	CCAACAACCTCTCCTTTTCAAATT	CTCTGATTTTGCTCATTCCTGAATAT	XM_003588826
MtGR	CCCAAGACGAATTTGTAAACACAT	CCCAATAAAAGAAACATACAAACAAGTC	BT149819
MtMR	CTACAGCTGGGGTCGTTCTT	AAGCCCTTAACCACAAATCG	Medtr7g034715
MtGalLDH	CTCCAAGCAAGGCTGAGAAA	GCTTTTCCAACTTGTTGTTCG	Medtr1g050360
MtG6PDH	ACAAGCCGGGAAGTAGAGGT	TGGTCACTCTATAAGGTAGGAGGAA	Medtr7g037440
Mt6PGDH	CGAGACTACTTCGGTGCTCA	TCTAAATCCTCGACTGCTTGG	Medtr7g017900
MtICDH	TGGCTGCTGATCTTAAAACAAA	TGCTTCGTTCCCCTTTCTTA	Medtr5g077070
MtCuZnSODa	TGGTTGGCTTGACTCCAGTA	AATGGCAGTAGCCCATCAAG	Medtr4g057240
MtCuZnSODb	CTCACCGGACCAAACTCAAT	AGCTACTCTGCCACCAGCAT	Medtr7g114240
MtCuZnSODc	GAAGGGCTGTCGTTGTTCAT	TCCAATGATACCGCATGCTA	Medtr6g029200
MtFeSOD	TCAATTCCAAATTACTGACTTTGTG	ATATTCAGGACGCCGATTCT	Medtr1g048990
MtMnSOD	TAAAGTGCTGCTTGGTGTGG	CTGCGCCCTAAGGAAAGTCT	Medtr3g094250
MtCAT	GCACCCGACAGGCAAGATAGATT	CGAGTCACGTACATGGAGTTTA	XM_013606823

MtACTIN11: actin; *VaP5CS*: *Vigna aconitifolia* Δ1-pyrroline-5-carboxylate synthetase; *MtP5CS1*: Δ1-pyrroline-5-carboxylate synthetase 1; *MtP5CS2*: Δ1-pyrroline-5-carboxylate synthetase 2; *MtP5CR*: Pyrroline-5-carboxylate reductase; *MtProDH*: proline dehydrogenase; *MtP5CDH*: Δ1-pyrroline-5-carboxylate dehydrogenase; *MtOAT*: ornithine δ-aminotransferase; *MtProT*: proline transporter; *MtCYS*: cysteine synthase; *MtECS*: γ- glutamyl-cysteine synthetase; *MtGSHS*: glutathione synthetase; *MthGSHS*: homoglutathione synthetase; *MtPCS*: phytochelatin synthase; *MtGPX*: glutathione peroxidase; *MtGR*: glutathione reductase; *MtMR*: monodehydroascorbate reductase; *MtGalLDH*: L-galactono-1,4-lactone dehydrogenase; *MtG6PDH*: glucose-6- phosphate dehydrogenase; *Mt6PGDH*: 6-phosphogluconate dehydrogenase; *MtICDH*: isocitrate dehydrogenase; *MtCuZnSODa*, *MtCuZnSODb*, *MtCuZnSODc*: cooper/zinc superoxide dismutases; *MtFeSOD*: ferric SOD; *MtMnSOD*: manganese SOD; *MtCAT*: catalase.
